# Fabrication of Stochastic Ni@PVP Nanowire Networks for Memristive Platforms

**DOI:** 10.3390/polym18060746

**Published:** 2026-03-19

**Authors:** Catarina Lemos, Catarina Dias, Rui S. Costa, João Ventura

**Affiliations:** IFIMUP—Instituto de Física de Materiais Avançados, Nanotecnologia e Fotónica, Departamento de Física e Astronomia, Faculdade de Ciências, Universidade do Porto, Rua do Campo Alegre s/n, 4169-007 Porto, Portugal

**Keywords:** electrochemical metallization, memristors, neuromorphic hardware, nickel nanowires, resistive switching

## Abstract

Single memristive nanowire networks have emerged as a promising pathway for energy-efficient neuromorphic computing, owing to their intrinsic nonlinearity, high dimensionality, fading memory and volatile switching dynamics relevant to physical reservoir computing. While prior works focused on oxide- or silver-based network systems, these approaches face trade-offs between operating voltage, cost, stability, and scalability. This work presents a proof-of-concept demonstration of stochastic polyvinylpyrrolidone (PVP)-coated nickel nanowire networks as low-cost and scalable memristive platforms, exhibiting low-voltage resistive switching (1–2 V). The electrical characterization reveals predominantly volatile resistive switching combined with nonvolatile behavior, consistent with a filamentary conduction mechanism at nanowire junctions. The switching dynamics are governed by the polymer coating thickness, with an intermediate PVP concentration (Ni@PVP = 1:25) showing optimal performance, with a resistance ratio of ~200, stable retention over 1 h, and a reproducible endurance of over 45 cycles. These results establish Ni@PVP nanowire networks as promising memristive platforms for neuromorphic hardware applications and physical reservoir computing, with relevant properties such as fading memory and nonlinear dynamics.

## 1. Introduction

Neuromorphic computing has emerged as a promising alternative to conventional von Neumann architectures, in which the physical separation between memory and processing units leads to massive power consumption in large-scale systems [1,2]. Neuromorphic systems shift the computational paradigm by drawing inspiration from the brain, which performs ~10^16^ operations per second while consuming only 20 W [3]. By emulating the structure and functionality of biological neurons and synapses, neuromorphic networks enable highly parallel and low-power computation [4].

Among neuromorphic paradigms, reservoir computing (RC) provides an efficient framework that can significantly reduce training and computational costs. In RC, a fixed nonlinear dynamical system, known as the reservoir, transforms inputs into a high-dimensional representation, while only a linear read-out layer is trainedto produce the output [5]. Interestingly, any physical system that exhibits nonlinearity, high dimensionality, fading memory and separation property can serve as a reservoir [6].

Within this context, memristive devices have gathered interest as artificial synapses for reservoirs due to their scalability, low-power operation, and simple structure [7,8]. Among RC architectures [9], self-assembled nanowire (NW) networks stand out due to their strong morphological and topological similarity to biological neuronal networks, exhibiting small-world connectivity, nonlinearity, separation property, fading memory, and high dimensionality [10]. Their neuromorphic behavior arises from filamentary resistive switching (RS) at NW junctions that act as artificial synapses. An insulating polymeric or oxide-based shell [11] enables the formation and rupture of conductive filaments, inducing SET transitions from a high-resistance state (HRS, R_OFF_) to a low-resistance state (LRS, R_ON_), and RESET in the reverse direction. The intrinsic stochastic morphology of the network leads to the formation of many junctions, resulting in rich high-dimensional and nonlinear dynamics.

To date, most memristive NW networks reported for physical RC rely on oxide-based systems [12,13], noble metals such as silver [14,15,16,17], and organic/organic nanowires like single-walled carbon nanotube (SWNT)/polyoxometalate (POM), SWNT/porphyrinpolyoxometalate (Por-POM), and SWNT/liquid crystals [18]. Oxide-based networks, such as Ni/NiO [13,19,20] and Cu/CuO core–shell NWs [14], show high R_OFF_/R_ON_ but require high SET voltages (>5 V) and rely on ordered structures [19,21]. In contrast, Ag-based NW networks, particularly Ag/polyvinylpyrrolidone (PVP), enable low-voltage RS in fully stochastic self-assembled architectures [16,17,22]. For instance, Milano et al. demonstrated in materia RC capable of temporal data processing, highlighting the potential of stochastic memristive systems as physical reservoirs [17,22]. However, Ag-based devices suffer from high cost, limited long-term stability, and scalability issues, motivating the search for alternative materials. Nickel emerges as a promising candidate due to its abundance, chemical stability, and compatibility with scalable chemical synthesis methods. Previous studies focused on ordered Ni/NiO core–shell NW networks, reporting SET values of 6–8 V in crossbar arrays [19] and similar behavior in magnetically aligned NW meshes [20]. Among polymer coatings, PVP is especially attractive, as it enables ionic transport and filament formation at NW junctions, leading to RS in metal NW networks [16,22].

In this work, we present a study of stochastic networks of PVP-coated nickel (Ni@PVP) NWs. Through comprehensive structural and morphological characterization using X-ray diffraction, Raman spectroscopy and scanning electron microscopy, combined with electrical analysis of switching behavior, volatility, retention, and endurance, we demonstrate that Ni@PVP NWs achieve competitive memristive performance, with low switching voltages (1–2 V) and R_OFF_/R_ON_~200. These findings establish Ni@PVP NW networks as a promising cost-effective and scalable platform for neuromorphic hardware implementations.

## 2. Materials and Methods

### 2.1. Synthesis of Ni NWs

Ni NWs were synthesized via a magnetically assisted simple chemical reduction method in a polyol medium [23,24]. Typically, 0.150 g of PVP (MW = 40,000 g mol^−1^ from Sigma-Aldrich, St. Louis, MO, USA) was dissolved in 30 mL of ethylene glycol (EG, 99% from Carlo Erba Reagents, Val-de-Reuil, France) followed by the dissolution of 0.036 g of nickel (II) chloride hexahydrate (NiCl_2_·6H_2_O, 98% from Sigma-Aldrich, St. Louis, MO, USA) in the same flask. After complete dissolution, 1 mL of hydrazine monohydrate (>98%, from Alfa Aesar, Haverhill, MA, USA) was added dropwise. The reaction was then placed in a pre-heated oil bath maintained at 85 °C, above a magnet for 30 min. Approximately 5 min later, a black precipitate began to form at the surface. At the end, the product was collected with a magnet and washed three times with ethanol to remove unreacted precursors. The obtained material was redispersed and stored in 10 mL of pure ethanol (>99%).

### 2.2. Fabrication of Ni NW Network

The fabricated single-coated Ni@PVP NWs were subsequently double-coated with PVP. This was achieved by dispersing the Ni NWs in freshly prepared PVP solutions dissolved in pure ethanol, with different Ni:PVP weight ratios (1:10, 1:25, 1:50), using a mechanical stirrer at 200 rpm for 6 h at room temperature. The resulting samples are referred to as Ni@PVP_SC_, Ni@PVP_1:10_, Ni@PVP_1:25_, and Ni@PVP_1:50_ NWs.

Ion beam deposition was used to fabricate Al (150 nm)/W (75 nm) thin films on glass substrates at a working pressure of 1.4 × 10^−4^ Torr (5 sccm of Ar flux; base pressure below 10^−6^ Torr). An array of two 30 µm wide electrodes separated by a 100 μm gap were then defined by photolithography. The photoresist (Microposit S1818 G2 photoresist from Atis S. A, DuPont, Wilmington, DE, USA) was spin-coated (2500 rpm, 35 s), pre-baked at 120 °C for 90 s, and exposed to UV light (365 nm, 48 s). Development was performed with a Microposit 351 developer from DuPon (Wilmington, DE, USA). Finally, memristive nanowire networks were fabricated by depositing the Ni NWs dispersed in pure ethanol at the electrode gaps by drop casting 4–5 drops (8 μL).

### 2.3. NWs and Network Characterization

The morphology of the NWs was characterized using scanning electron microscopy (SEM) with a high-resolution scanning microscope FEI Quanta 400 FEG ESEM (FEI Company, Hillsboro, OR, USA) using 15 kV in both scattering and backscattered electron mode. Structural analysis was performed using X-ray diffraction (XRD) with a SmartLab Rigaku diffractometer (Rigaku Corporation, Tokyo, Japan) using Cu K_α_ radiation (λ = 1.5404 Å) in the Bragg–Brentano *θ*/2*θ* configuration with a range of 10° to 90°, with a step of 0.01°. Composition was obtained using Raman spectra at room temperature with a Renishaw inVia Qontor Spectrometer (Renishaw plc, Wotton-under-Edge, United Kingdom) in the 200–3500 cm^−1^ spectral range using a 532 nm edge He-Ne laser line at 1.7 mW and a 50× magnification lens, with 20 s of exposure and 1% power for 3 accumulations. The electrical behavior of the networks was characterized at room temperature using two tungsten microprobes and a 2410 Keithley SourceMeter (Keithley Instruments, Cleveland, OH, USA), applying voltage cycles from 0 V → +V_max_ → 0 V → −V_max_ → 0 V. Retention measurements were performed for over 1 h by applying low-voltage read pulses (3–5 mV) under a current compliance of 0.1–1 mA. Endurance was determined through repeated pulsed switching (+5 V and −1 V).

## 3. Results

### 3.1. Structure Characterization

**Figure 1:**
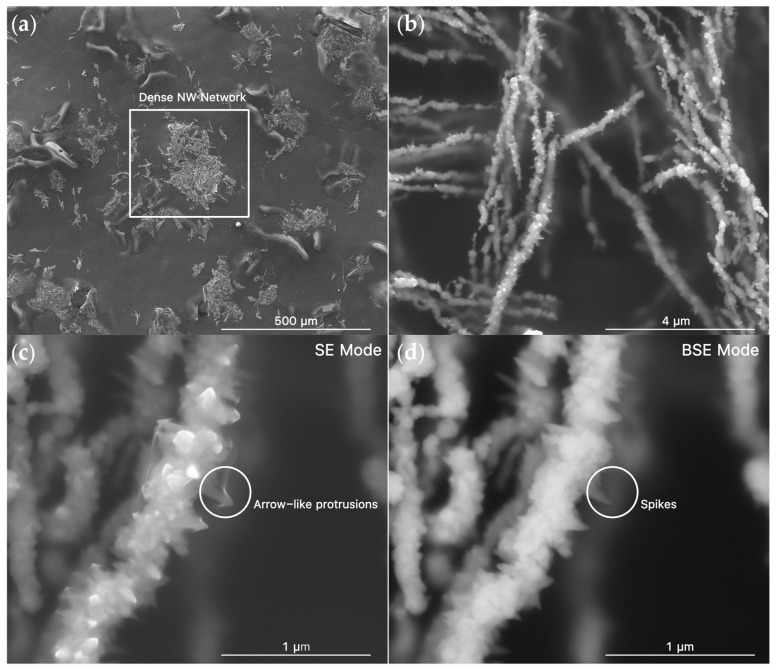
SEM images of Ni@PVP_1:10_ at (**a**) low (200×) and (**b**) high (25,000×) magnifications for a dense NW network. SEM image of the surface of a single NW at a magnification of 100,000× for (**c**) secondary and (**d**) backscattered electron modes.

To investigate the influence of polymer coating concentration on RS, the fabricated Ni NWs were double-coated at different Ni:PVP weight ratios (1:10, 1:25, and 1:50). SEM confirmed the formation of well-defined Ni NWs (Figure 1a), with single-coated NWs with an average diameter of 209 ± 26 nm and length of 23 ± 14 μm ranging from 5 to 85 μm. These dimensions were consistent across the different ratios of double coating (Figure S1). The Ni@PVP_1:10_ NWs exhibit a characteristic spiky surface (Figure 1b) and a surrounding coating layer. In secondary electron image, these shapes appear as arrow-like protrusions (Figure 1c), while, in comparison, the corresponding backscattered electron image (Figure 1d) reveals triangular structures extending from the NW surface. This indicates that the arrow-like shapes in secondary electron mode arise from imaging distortions associated with the polymer coating layer rather than from the metallic NW surface itself, proving the successful coating [25]. No significant morphological differences were observed between the NWs prepared with and without the double-coating process at different PVP ratios (Figure S2). In all cases, the NWs form a highly interconnected stochastic network with multiple junctions, revealing that the network architecture is preserved after the double-coating process.

**Figure 2:**
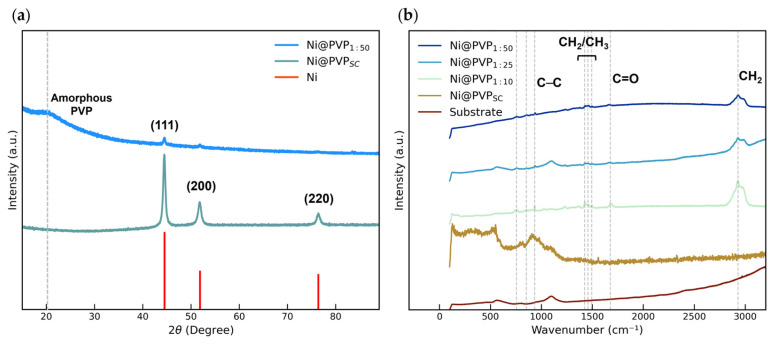
(**a**) XRD diffractograms of Ni@PVP_SC_ and Ni@PVP_1:50_. Ni and amorphous PVP peaks are highlighted with vertical lines. (**b**) Raman spectra of Ni NWs coated with different concentrations of PVP (single coated, 1:10, 1:25, 1:50). PVP modes are highlighted with vertical lines.

Figure 2a shows the XRD diffractograms of Ni@PVP_SC_ and Ni@PVP_1:50_ NWs, confirming the presence of metallic Ni through peaks at 45°, 52° and 76° corresponding to the (111), (200), and (220) planes of the face-centered cubic (FCC) crystal structure, respectively (JCPDS card 00-04-0850) [26,27]. For the highest PVP concentration (1:50), the additional broad peak at 20° is attributed to amorphous PVP resulting from increased polymer content [28,29].

Figure 2b shows the Raman spectra of single- and double-coated Ni NWs. Vibrational bands at 2928, 1660, 1495, 1464, 1448, 1426 and 1376 cm^−1^ are consistent with pure PVP [27], and their intensity increases with polymer content. Notably, the band at 2928 cm^−1^ corresponds to the asymmetric CH_2_ stretching in the PVP backbone, indicating proximity of the polymer chain to the Ni NW surface [30]. The strong peak at ~1600 cm^−1^ is assigned to the PVP C=O stretch, while the vibrational bands between 1370 and 1500 cm^−1^ arise from CH_2_/CH_3_ deformation modes and PVP ring/C–N skeletal vibrations [31]. Together with XRD, these results confirm the presence of PVP and show that higher polymer content enhances the amorphous contribution, supporting the interpretation that the low-contrast surrounding layer observed in SEM images corresponds to the polymer coating.

### 3.2. Electrical Characterization

**Figure 3:**
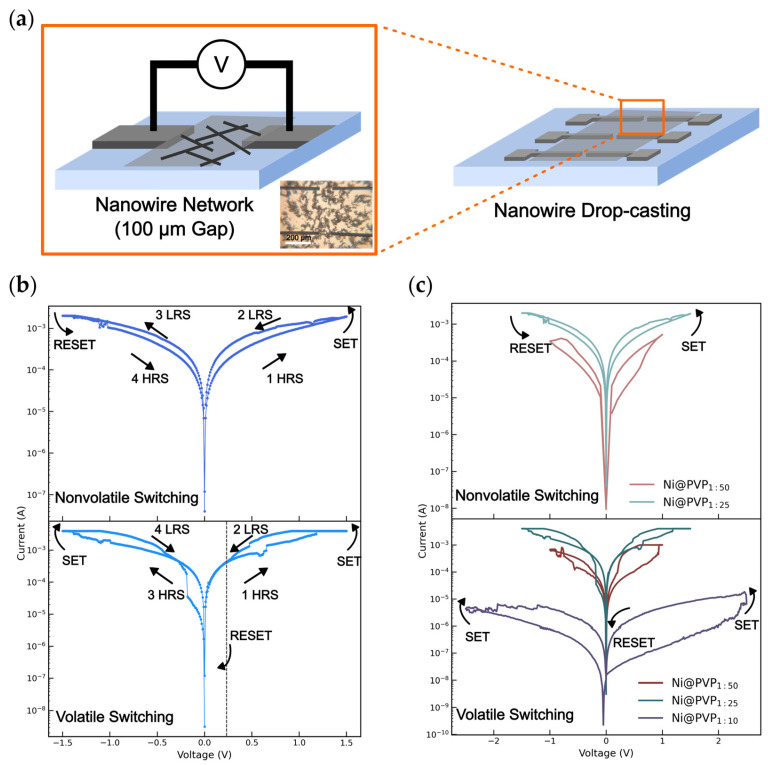
(**a**) Schematic of the networks. The inset shows the optical image at one of the gaps. *I–V* curves for Ni@PVP_1:25_ device showing (**b**) nonvolatile and volatile RS. The gray line at 0.2 V represents the voltage where the volatile device relaxes to HRS. (**c**) Characteristic *I–V* curves for Ni@PVP nonvolatile and volatile devices with different PVP ratios of 1:10 (purple), 1:25 (green) and 1:50 (red). The arrows show the sweeping direction.

To assess the electrical properties of the Ni@PVP NWs, stochastic networks were drop-casted in between Al/W electrodes (Figure 3a). The Ni@PVP_SC_ device shows linear ohmic behavior (Figure S3), confirming that a single synthesis-derived PVP layer provides insufficient NW insulation to support RS. The double-coated Ni@PVP_1:25_ and Ni@PVP_1:50_ devices exhibited both nonvolatile and volatile RS. This coexistence reflects the inherent stochastic nature of the network architecture, in which local variations in NW junction geometries and polymer thickness produce distinct switching behaviors across different current pathways. A representative nonvolatile *I–V* characteristic curve for Ni@PVP_1:25_ is shown in Figure 3b. Initially, the network is in an HRS with a resistance of 1.4 × 10^3^ Ω. Upon increasing the applied voltage up to +1.5 V, a SET occurs switching it to a LRS of 674 Ω. When the voltage is reduced and swept through zero bias, the LRS is retained, demonstrating nonvolatile behavior. During the reversed polarity voltage sweep, the electrical resistance switches back to HRS (RESET) at −1.1 V.

Under the same procedure, the Ni@PVP_1:25_ network also exhibits volatile RS behavior (Figure 3b). Upon increasing the voltage, the network shows a progressive increase in current and transitions to a LRS of 230 Ω at around 1.2 V. As the applied voltage approaches zero, the device spontaneously relaxes back to the HRS of 5.2 × 10^3^ Ω at around 0.2 V (volatile behavior). This indicates the formation of unstable conductive filaments that are only sustained under an applied electric field and dissolve with low voltages, likely due to ionic diffusion of the metal ions within the polymer layer. A similar volatile response is observed under negative polarity. Interestingly, the same NW network can exhibit nonvolatile switching during the first cycles before evolving into a dominant volatile regime. This suggests that, although conductive filaments can initially stabilize with the trapping of ions, continuing cycles promote filament instability, likely due to localized Joule heating and the degradation of the insulating layer at NW junctions [32,33]. Figure 3c compares the influence of polymer coating on RS. All devices show RS, with Ni@PVP_1:25_ and Ni@PVP_1:50_ exhibiting both volatile and nonvolatile behaviors. However, networks with the lowest PVP concentration Ni@PVP_1:10_ showed over one order of magnitude lower currents and a volatile response only. Although a reduced polymer coating would be expected to increase the current due to a thinner insulating layer, a previous study [34] has shown that thin switching layers in filamentary devices promote the formation of narrower conductive filaments. The smaller filament cross-section limits the current, as fewer ions are required to bridge the electrodes, resulting in lower operating currents. These results highlight the role of PVP concentration in governing ionic transport and filament formation at NW junctions.

**Figure 4:**
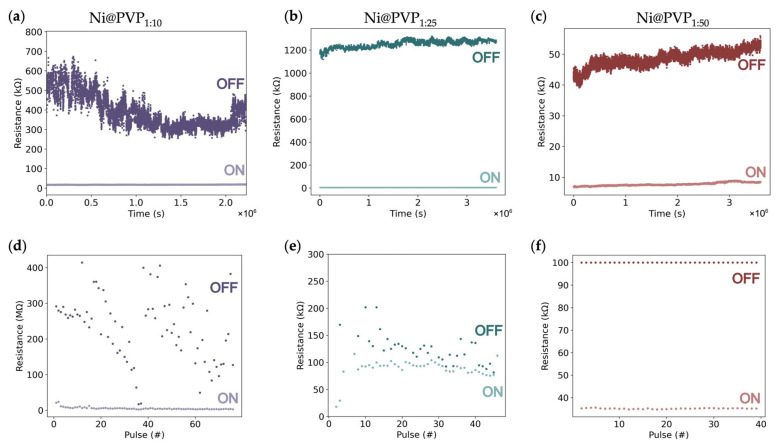
Retention behavior for Ni@PVP NW network devices, at a reading voltage of 3–5 mV for (**a**) Ni@PVP_1:10_, (**b**) Ni@PVP_1:25_, and (**c**) Ni@PVP_1:50_. Endurance with pulsed voltage for (**d**) Ni@PVP_1:10_ (+3 V for SET and −5 V for RESET, 100 ms width), (**e**) Ni@PVP_1:25_ (+5 V for SET and −1 V for RESET, 100 ms width), and (**f**) Ni@PVP_1:50_ (+5 V for SET and −1 V for RESET, 100 ms width).

Retention was monitored for over 1 h by applying voltage read pulses of 3–5 mV under a current compliance of 0.1–1 mA (Figure 4a–c). A slight decay of the OFF resistance was observed for the Ni@PVP_1:10_ device (Figure 4a). This degradation could be associated with partially irreversible ion distribution in the PVP layer [35]. Ni@PVP_1:10_ devices exhibited a lower ratio (~25), and the resistance varied significantly over the 1 h measurement (Figure 4a) due to the OFF state showing large fluctuations. The Ni@PVP_1:50_ devices also showed a low resistance ratio (~8.5) but comparatively smaller resistance fluctuations (Figure 4c), which could indicate that excessive PVP content increases the effective barrier thickness suppressing filament formation. In contrast, the intermediate Ni@PVP_1:25_ devices exhibited the highest resistance ratio (~200) with the most stable retention over time, demonstrating an optimal polymer concentration for stable filament formation and thus reproducible switching (Figure 4b).

The endurance measurements further corroborate these observations (Figure 4d–f). Ni@PVP_1:10_ devices showed poor endurance performance with large HRS fluctuations (Figure 4d). On the other hand, both Ni@PVP_1:25_ and Ni@PVP_1:50_ devices exhibited more-stable switching, with Ni@PVP_1:50_ having a better performance for over 40 pulses (Figure 4e and Figure 4f, respectively). These results indicate that the switching dynamics in Ni@PVP devices are sensitive to the polymer concentration, as the polymer controls the filament length, rupture dynamics and ionic migration [10].

**Figure 5:**
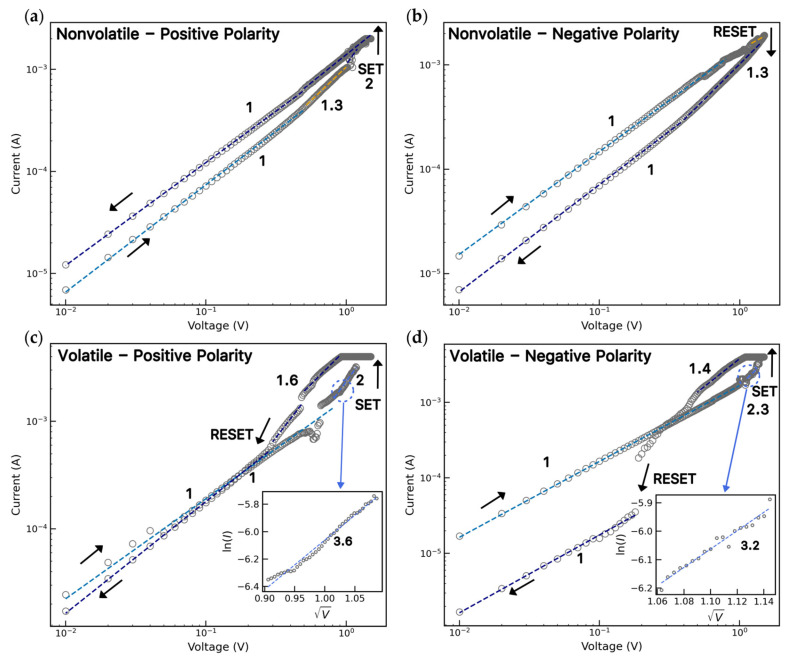
Log–log plots of *I–V* curves for the (**a**) SET and (**b**) RESET in nonvolatile Ni@PVP_1:25_ device. (**c**) Positive and (**d**) negative polarity in volatile Ni@PVP_1:25_. The insets show the plots in $\ln\;(I)\;\mathrm{vs}.\;\sqrt{V}$ with their respective slopes.

To investigate the dominant conduction mechanism underlying the RS behavior, the *I–V* curves were analyzed on a log–log scale (Figure 5). During the SET of the nonvolatile network (Figure 5a), two distinct conduction regimes were observed. At low applied bias, the current increases linearly with voltage (slope ~1), indicating an ohmic conduction [36,37] governed by thermally assisted carrier transport and the initial migration of metal ions through the insulating PVP layer. As the applied bias increases, the conduction transitions to a space-charge-limited current (SCLC) mechanism (slopes of ~1.3 and 2), wherein injected carriers progressively fill trap states [38,39]. Once the traps are filled, a sudden increase in current occurs, corresponding to the formation of a conductive filament and the transition to the LRS (SET). In the LRS, the slope returns to ~1 at higher currents, consistent with metallic conduction. Under reverse bias (Figure 5b), the ohmic behavior is initially maintained and deviates as the voltage increases, near the RESET region (slope of 1.3–1.5), followed by an abrupt decrease in current associated with filament rupture and the return to the HRS. The volatile RS follows an identical sequence of conduction mechanisms (Figure 5c,d), although RESET occurs before the applied voltage reaches 0 V for both positive and negative polarities. Both resistive switching behaviors are consistent with electrochemical metallization mechanisms previously reported for metal oxide- and Ag-based memristive devices [19,20,22].

The prevalence of volatile RS, despite possible ion trapping in the PVP layer, shows the role of interfacial effects in these stochastic networks. Localized Joule heating at the NW junctions can induce structural changes and charge injections into the PVP layer, destabilizing the conductive filaments. In the pre-SET region of the volatile response (insets in Figure 5c,d), the charge transport is dominated by Schottky emission, as evidenced by the linear fit of $ln\;(I)\;vs.\sqrt{V}$ [40,41]. This behavior is consistent with thermionic emission, where electrons gain sufficient thermal energy, facilitated by localized Joule heating at the NW junction, to pass the interfacial potential barrier of the PVP layer [42,43]. Consequently, the conductive filaments formed under bias are unstable and dissolve spontaneously upon voltage reduction, leading to the observed volatile response [43]. The transition from initial nonvolatile to dominant volatile switching likely arises from the progressive thermal modification of the junctions of the stochastic NW network over repeated cycling.

**Figure 6:**
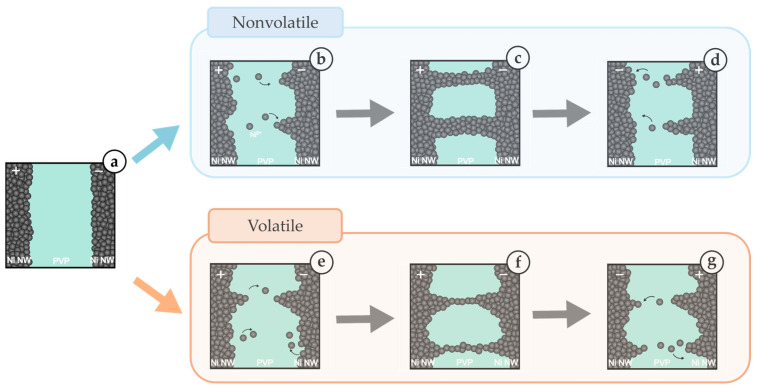
Schematic illustration of the filamentary RS mechanism in nonvolatile (**top**) and volatile (**bottom**) Ni@PVP NW memristive networks. (**a**) Ni@PVP network initially in HRS, (**b**,**e**) upon increasing the applied bias, anodic dissolution of nickel creates Ni^2+^ ions that migrate through the PVP, initiating the formation of conductive filaments. (**c**,**f**) The complete formation of conductive filaments (LRS) corresponds to the SET. (**d**,**g**) By decreasing the applied bias, the filaments suffer dissolution with ions drifting back to nickel cores (HRS).

Figure 6 illustrates the RS mechanism observed in Ni@PVP NW networks inferred from electrical characterization [9,37]. RS occurs at the PVP junctions between contacting NWs, where each nickel core acts as an electrode. For volatile devices, initially, the Ni@PVP NW network is in the HRS. Under an applied bias the anodic dissolution of nickel occurs, generating nickel ions (Ni → Ni^2+^ + 2e^−^), which migrate through the PVP layer (Figure 6a,b). These ions accumulate and form a conductive filament at the junctions between nanowires, creating a percolative conduction path within the network, which leads to the SET (Figure 6c). Upon reduction or removal of the applied bias, unstable filaments may partially or fully dissolve due to ionic diffusion, resulting in relaxation back to the HRS and volatile RS (Figure 6d). The volatile nature of the switching response shapes the filament formation in the middle since it can be achieved by applying both negative and positive bias voltages [44,45].

In contrast, nonvolatile devices exhibit thicker and more-stable conductive filaments, which require reverse polarity to induce rupture. Thin coatings may promote the formation of less-stable filaments, whereas excessively thick coatings hinder ionic transport and lead to weak or incomplete filament formation, suggesting the existence of an optimal coating ratio. Moreover, some degree of coating non-uniformity across the network may contribute to the observed variability, enabling the coexistence of both nonvolatile and volatile switching within the same network. This qualitative behavior was consistently observed across several fabricated devices for each Ni:PVP ratio, including from repeated syntheses, indicating the reproducibility of the method. Although such transition may raise concerns regarding device stability in conventional memory applications, it can be advantageous for unconventional computing approaches such as reservoir computing [46].

The key RS properties of the fabricated memristive NW networks are summarized and compared with those of other reported structures in Table 1. The demonstration of stable RS at 1–2 V in Ni NW networks marks a clear advance over conventional oxide-based Ni/NiO structures, which typically operate at higher voltage values (4–5 V) [19]. This low-voltage performance positions the fabricated Ni NW devices alongside Ag-based platforms, widely recognized as the state-of-the-art in filamentary switching due to their efficient operation [16,17,22]. These devices also exhibit a R_OFF_/R_ON_ of ~200 (for the 1:25 sample), comparable with Ag/AgO_x_ systems (~100) [15]. Although Ni/NiO structures can reach higher ratios (>10^5^) [19], they generally rely on ordered architectures. The endurance >40 cycles, while lower than the 100–1000 cycles reported for oxide-based and Cu/TiO_2_ systems [19], remains comparable with volatile Ag/AgO_x_ devices (~70 cycles) [15]. Furthermore, Ni provides a more sustainable and cost-effective alternative, compatible with scalable chemical fabrication routes [47,48]. The template-free chemical reduction process for the synthesis of the Ni NWs can be performed without the need for complex or specialized equipment, unlike other fabrication methods such as chemical vapor deposition or electrochemical deposition [24]. In addition, the stochastic self-assembly nature of the networks by drop casting is compatible with large-area fabrication. These characteristics make Ni@PVP networks a promising and potentially scalable platform for low-cost neuromorphic hardware.

## 4. Conclusions

Herein, a systematic study of stochastic memristive Ni@PVP NW networks was performed, as a cost-effective and scalable alternative to precious-metal and oxide-based memristive platforms. The synergy between the chemically stable and abundant Ni cores and the controlled polymer coating enables a self-assembled NW network that operates at low voltages while maintaining competitive electrical performance. PVP coating plays a crucial role in regulating ion diffusion and filament formation, with an intermediate Ni@PVP ratio of 1:25 yielding the optimal performance. In contrast, insufficient coating (1:10) leads to unstable, predominantly volatile switching with low resistance ratios, while excessive coating (1:50) hinders ion migration and degrades switching performance. These results establish polymer thickness as a key design parameter for tuning memristive behavior in metal NW networks. The Ni@PVP NW networks operate at 1–2 V, which is a significant improvement over conventional Ni/NiO-based devices (4–5 V) and comparable to state-of-the-art Ag-based platforms, with a resistance ratio of ~200, stable retention over 1 h and endurance over 45 pulses. The switching mechanism is consistent with electrochemical metallization at NW junctions, in which the coexistence of volatile and nonvolatile responses reflects the stochastic variability of the network architecture. Notably, the predominant volatile switching provides intrinsic fading memory behavior which, together with the nonlinearity and high-dimensional dynamics arising from the stochastic network topology, positions Ni@PVP NW networks as promising practical platform for neuromorphic hardware applications.

## Figures and Tables

**Table 1:** Overview of the memristive properties of NW networks for reservoirs.

Network	Voltage (V)	Type	Retention	$R_{OFF}/R_{ON}$	Endurance (Pulses)	Ref.
Ni@PVP_1:10_	1–2	Mixed	1 h	25	Poor	This work
Ni@PVP_1:25_				200	45	
Ni@PVP_1:50_		Volatile		8.5	40	
Ag/Ag_2_S atomic switches	1.5	Volatile	-	-	-	[49]
Ag/AgO_x_	2	Volatile	-	100	35	[15]
Ti/Au/Ag/PVP planar NW network	1–8	-	-	-	-	[16]
Au/Ti/Ag/TiO_2_ planar NW network	25–100	Volatile	-	-	40	[12]
Au/Ti/Ag/TiO_2_ planar NW network	19	Volatile	-	-	-	[50]
Ag/ZnO mesh	4	-	-	10^5^	9	[21]
Ni/Ag/PVP	20	-	-	-	-	[51]
Ni/NiO	3	-	-	>10^5^	20	[13]
Ti/Au/Ni/NiO crossbar NWs	5	Nonvolatile	>24 h	10^7^	10	[19]
Ti/Au/Ni/NiO mesh	5	-	-	-	10	[20]
Ti/Cu/TiO_2_	0.6	-	-	574	100	[52]

## Data Availability

The raw data supporting the conclusions of this article will be made available by the authors on request.

## Supplementary Materials

The following supporting information can be downloaded at https://www.mdpi.com/article/10.3390/polym18060746/s1: Figure S1: Length and diameter distributions of Ni@PVP nanowires; Figure S2: SEM images of Ni@PVP NW networks at different magnifications; Figure S3: *I–V* characteristic curves of single-coated Ni@PVP.

## Abbreviations

The following abbreviations are used in this manuscript:

HRS	High-Resistance State
LRS	Low-Resistance State
NW	Nanowire
PVP	Polyvinylpyrrolidone
RC	Reservoir Computing
RS	Resistive Switching
SE	Secondary Electrons
SEM	Scanning Electron Microscopy
XRD	X-ray Diffraction
