# Supplementary material for: Fabrication of Stochastic Ni@PVP Nanowire Networks for Memristive Platforms
Source: Polymers (Basel). 2026 Mar 19;18(6):746. doi: 10.3390/polym18060746 (PMC13030839; doi:10.3390/polym18060746)
Supplement: Supplementary file 1 [file polymers-18-00746-s001.zip › polymers-4206680-supplementary.pdf]

Fabrication of Stochastic Ni@PVP Nanowire Networks for Memristive Platforms

Catarina Lemos, Catarina Dias, Rui S. Costa * and João Ventura

IFIMUP—Instituto de Física de Materiais Avançados, Nanotecnologia e Fotónica, Departamento de Física e Astronomia, Faculdade de Ciências, Universidade do Porto, Rua do Campo Alegre s/n, 4169-007 Porto, Portugal

*Correspondence: rucosta@fc.up.pt

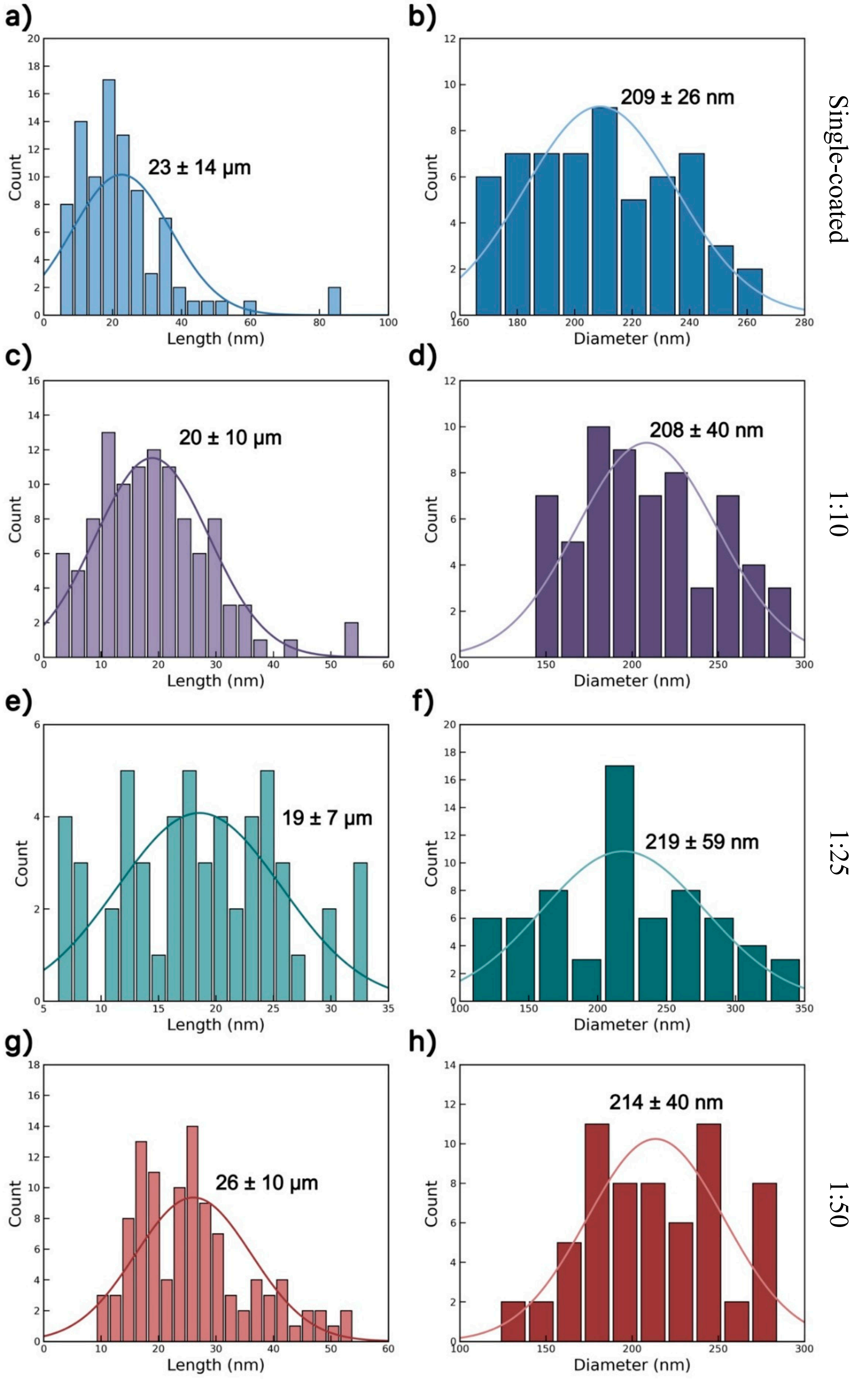

Figure S1: Length and diameter distributions of Ni@PVP NWs for (a-b) Ni@PVP$_{SC}$, (c-d) Ni@PVP$_{1:10}$, (e-f) Ni@PVP$_{1:25}$, and (g-h) Ni@PVP$_{1:50}$. Data calculated from SEM images using ImageJ.

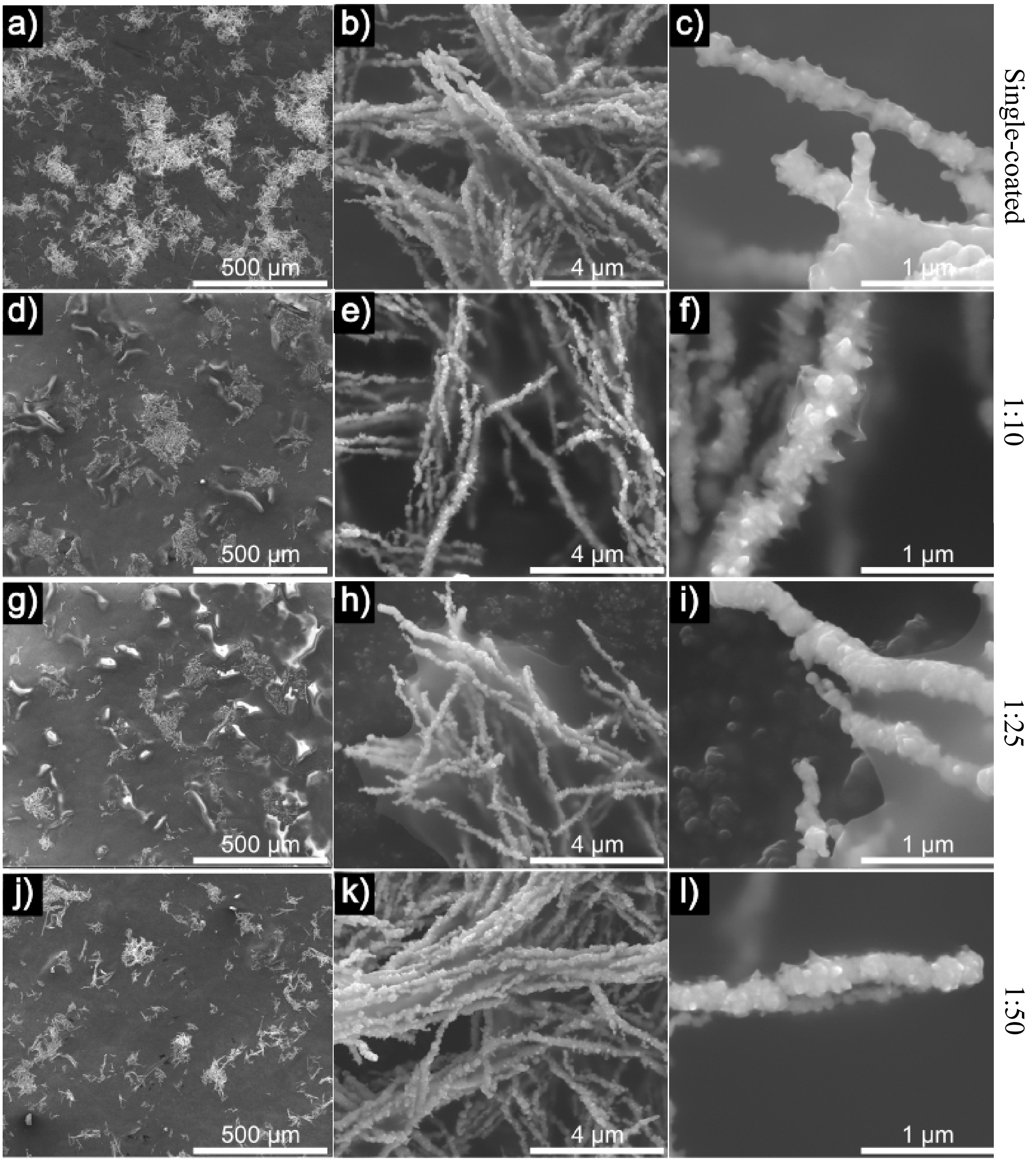

Figure S2: SEM images of Ni@PVP NW networks for (a-c) $Ni@PVP_{SC}$, (d-f) $Ni@PVP_{1:10}$, (g-i) $Ni@PVP_{1:25}$, and (j-l) $Ni@PVP_{1:50}$ acquired at 200×, 25 000× and 100 000× magnifications, respectively.

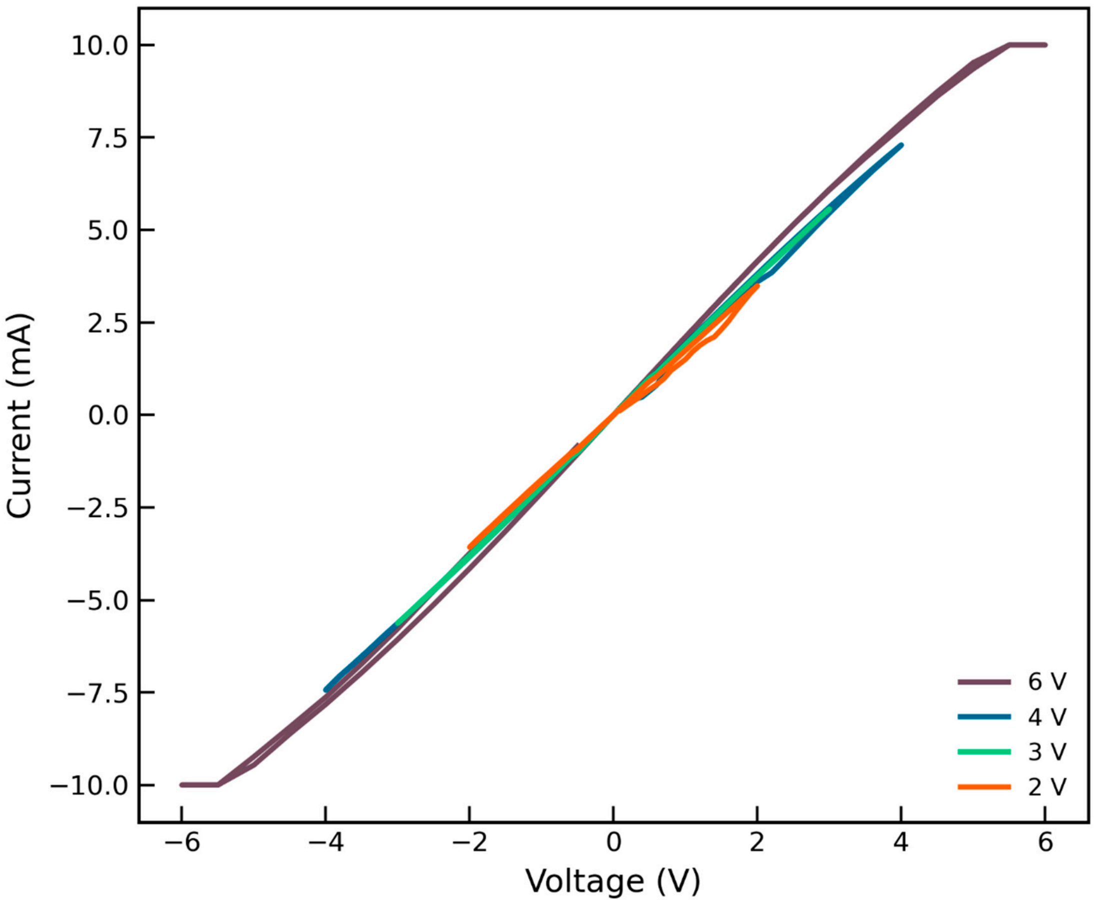

Figure S3: *I-V* characteristic curves of single-coated Ni@PVP for 2–6 V.
